# The future of the global food system

**DOI:** 10.1098/rstb.2010.0180

**Published:** 2010-09-27

**Authors:** H. Charles J. Godfray, Ian R. Crute, Lawrence Haddad, David Lawrence, James F. Muir, Nicholas Nisbett, Jules Pretty, Sherman Robinson, Camilla Toulmin, Rosalind Whiteley

**Affiliations:** 1 Department of Zoology and Institute of Biodiversity at the James Martin 21st Century School, University of Oxford, South Parks Road, Oxford OX1 3PS, UK; 2 Agriculture and Horticulture Development Board, Stoneleigh Park, Kenilworth, Warwickshire CV8 2TL, UK; 3 Institute of Development Studies, Falmer, Brighton BN1 9RE, UK; 4 Syngenta AG, PO Box CH-4002, Basel, Switzerland; 5 Institute of Aquaculture, University of Stirling, Stirling, Stirlingshire FK9 4LA, UK; 6 Foresight, UK Government Office for Science, 1 Victoria Street, London SW1H OET, UK; 7 Department of Biological Sciences, University of Essex, Wivenhoe Park, Colchester, Essex CO4 3SQ, UK; 8 International Institute for Environment and Development, 3 Endsleigh Street, London WC1H 0DD, UK

**Keywords:** food security, food system, population growth, consumption growth, agriculture, climate change

## Abstract

Although food prices in major world markets are at or near a historical low, there is increasing concern about food security—the ability of the world to provide healthy and environmentally sustainable diets for all its peoples. This article is an introduction to a collection of reviews whose authors were asked to explore the major drivers affecting the food system between now and 2050. A first set of papers explores the main factors affecting the demand for food (population growth, changes in consumption patterns, the effects on the food system of urbanization and the importance of understanding income distributions) with a second examining trends in future food supply (crops, livestock, fisheries and aquaculture, and ‘wild food’). A third set explores exogenous factors affecting the food system (climate change, competition for water, energy and land, and how agriculture depends on and provides ecosystem services), while the final set explores cross-cutting themes (food system economics, food wastage and links with health). Two of the clearest conclusions that emerge from the collected papers are that major advances in sustainable food production and availability can be achieved with the concerted application of current technologies (given sufficient political will), and the importance of investing in research sooner rather than later to enable the food system to cope with both known and unknown challenges in the coming decades.

## 1. Introduction

The supply and availability of food has been a crucial factor shaping the emergence, development and persistence of human civilizations throughout the ages. For the last few decades food has been cheaper in real terms, and more readily available, than probably at any time in history, which partly explains why food policy has received less prominence in national and international decision-making than in earlier times. Yet, we cannot be said to have a functioning global food system when one in seven people today still do not have access to sufficient food, and an equal number are over-fed. Looking ahead, we can identify known threats to the food system and factors that will increase the risks of a rise in hunger. Population and consumption growth will lead to the demand for food increasing for most of the current century, while increasing competition for land, water and other resources threaten the supply of food. Overarching this is the threat of global change, and the needs to make the food system resilient to shocks that cannot be predicted in advance. As the reactions to the 2008 spike in commodity prices presaged, food policy is likely to increase in importance in the coming decades.

The authors of the reviews in this issue were asked to explore the main drivers of change affecting the global food system between now and 2050. The reviews were commissioned as part of the UK's Government Office for Science Foresight project on *Global Food and Farming Futures*, which will report in late 2010.^1^ The project is based around the organizing question of how a future global population of 9 billion people can all be fed healthily and sustainably, and the reviews contribute towards an evidence base underlying an exploration of the policy options to address these challenges. Science has a major role to play in feeding the world, as the green revolution amply demonstrated. But questions of food security require a multi-disciplinary approach involving the social sciences and economics; hence, the authors of the reviews are drawn from a broad range of disciplines.

We have organized the reviews into four sections, though there is some overlap. First we explore factors affecting the demand for food with four reviews on population growth, changes in consumption patterns, the effects of urbanization on the food system and the importance of understanding income distributions. The second section examines the supply side of the equation with reviews of likely changes in crop and livestock production, and the different components of aquatic food—marine capture, freshwater capture and aquaculture. The third section explores exogenous factors affecting the food system: the possible effects of climate change, competition for water, energy and land, and agriculture's dependency on, and provision of, ecosystem services. The final section explores cross-cutting themes, many involving economics: what is the relationship between productivity and investment in research and extension? How do we model the food system? What are the consequences of globalization and of volatility? This section also considers the issue of reducing waste in the food system and the interrelationship between diet and health.

## 2. Drivers of demand

Understanding trends in population size are critical to estimating the future demand for food. Lutz & Samir (2010) review how reliable population projections are now constructed. Populations in different countries are assumed to be composed of different age cohorts of the two sexes that vary in demographic rates such as mortality and fertility. Models can be extended to include differences between rural and urban populations (connected by migration) and, most importantly, educational status. There is very convincing evidence of the critical importance of female education and access to contraception in causally affecting fertility, and these are probably the chief mechanisms behind the decline in fertility as countries develop economically and go through the demographic transition. Of particular relevance here is evidence that education rates are also negatively correlated with malnutrition and food insecurity (Lutz *et al*. 2004). How to deal with uncertainty is a perennial problem in population and other forecasting and of the four strategies listed: ignoring it, constructing scenarios; exploring a plausible range of variation; and making fully probabilistic projections; Lutz & Samir strongly argue for the last. Though there is a risk that probabilistic projections appear spuriously precise, all assumptions are made rigorously specific. Interestingly, imprecision about the state of current populations can be as big a source of error as uncertainty about the future.

In studying future food demands, Lutz & Samir recommend adopting, with some caveats, the United Nations Population Division projections with the ‘medium fertility’ assumption (United Nations 2004). However, they argue that the UN projection of approximately 1.8 children per adult female overestimates fertility in China which is more likely to be 1.5 (though not as low as the official estimates of 1.2): China is big enough that this assumption makes a difference. With this adjustment, global population growth is predicted to decelerate and reach just over 9 billion in 2050. There are marked regional variations: Europe's population will decline, Africa's will double, while China will peak in about 2030 and be overtaken by India around 2020. Populations will age almost everywhere, but as the old will be healthier, rethinking age in terms of time to expected death (rather than time since birth) may give a different and more positive perspective on increased longevity.

Increasing demand for food is caused not only by a rise in population size but, as Kearney (2010) explores, a rise in *per capita* consumption. As people who are initially undernourished obtain access to more food calories, they first go through an expansion phase where diets contain more food—typically, grains, roots, tubers and pulses—and then a substitution phase, where the latter are replaced by more energy-rich foods such as meat and those with a high concentration of vegetable oils and sugar. The result is the nutritional transition (Popkin 1998), which has major implications for food supply as typically the production of high-energy food requires more resources (for example, instead of grain being directly consumed by humans, it is used as animal feed for livestock production which is then consumed by humans, overall a more inefficient process). Increased consumption of high-energy foods can increase the risk of obesity and the chronic diseases associated with being overweight: indeed, some countries that are still coping with under-nutrition in parts of their population are now suffering the additional burden of over-nutrition. The overall pattern of the nutritional transition hides many interesting local variations. For example, while China has seen a very strong increase in the consumption of high-energy foods, in India for cultural and religious reasons, the rise has been much less marked, for equivalent levels of income.

Global dietary patterns are also being influenced by a complex web of socio-economic trends and drivers. On the demand side, more and more people live in cities where they have relatively sedentary occupations and often have relatively high disposable incomes. On the supply side, economic growth, regulatory liberalization, the encouragement of foreign direct investment and globalization in general has allowed a burgeoning fast-food and supermarket sector to develop. As Kearney describes, in the 10 years between 1990 and 2000, the service and retail sectors in Latin America made changes that had previously taken 50 years in North America, and much of Asia and Eastern Europe are only a few years behind. This increased economic activity in the food sector brings advantages such as employment and investment opportunities, and often increases the availability and safety of the food on offer to its consumers. But by making cheap foods rich in fats and sugars easily available (many processed foods are 30% fat), there are important health implications. Significantly, South Korea, which has vigorously promoted local foods rather than a western diet, has lower rates of obesity than similar countries (Kennedy *et al*. 2004).

The implications of urbanization are explored in more detail by Satterthwaite *et al*. (2010). They point out that around 1940, the global economic value of industry and services for the first time exceeded that of the primary sector (food production, forestry and mining), and that by about 1980 more people were employed in the former than the latter. Industry and services are concentrated in cities and by about 2008 more people on the Earth lived in cities than in rural areas. This trend towards urbanization is certain to continue and the last few decades have seen the rise of megacities in developing countries, with Mumbai, Sao Paulo and Mexico City all having more than 16 million inhabitants. Urbanization is strongly associated with increasing wealth, and sufficiently advanced logistics and infrastructure are essential to feed the inhabitants in very large conurbations.

Increasing urbanization and urban spread has a direct, though sometimes, exaggerated effect on the land available for agriculture but many more indirect effects. Urban populations can access a greater diversity of foods, though this may include meat, dairy and convenience foods—types of food that may have required more resources for their production or be less healthy. However, some studies suggest that income rather than urban or rural location is normally the primary determinant of diet. Urbanization can also have very positive effects on rural areas and food production, in general by increasing national wealth and more specifically by creating markets for food producers. In developing countries in particular there are often strong financial links between people living in cities and the countryside, with remittances from urban households financing innovation and yield growth in farming.

To accurately predict a nation's food demand, it is important to know the full distribution of *per capita* income and how this is reflected in food purchases, an area of active research in behavioural economics. There is a lower limit to the amount of food an individual can eat without starving to death and an upper limit determined by our physiology. These biological facts underlie Engel's law, which states that as income increases the proportion spent on food declines. There are numerous challenges to estimating this relationship: moving from micro- to macro-economic description—from the behaviour of individuals to the aggregate properties of a population—is complicated by the nonlinearity of the Engel curve, what economists call the aggregation problem. One also cannot simply work with *per capita* behaviours, as household size and in particular the number of children affects the income–food demand relationship. Not all food requires the same resources for its production and to understand the full consequence of increased wealth, there is a need to couple Engel's Law with Bennett's Law, the latter describing the shift from starchy staples to more fatty foods as people get richer.

Cirera & Masset (2010) explore how these problems have been tackled in the economics literature and how they have been incorporated into the major general and partial equilibrium models that are used to model future food supply. They show how income disparity can affect estimates of future food demand, the nub of the aggregation problem. Most major models do not explicitly allow for this variation. To illustrate their arguments, the authors explore how changes in income distribution affect food demand in an Indian state, and then cautiously extrapolate their results to global food supply. Not taking into account income distribution may affect estimates of food demand, though perhaps by only approximately ±10 per cent.

## 3. Supply-side drivers

Crop yields have improved dramatically over the last 50 years, but there is evidence that rates of increase have declined in recent times. Jaggard *et al*. (2010) ask if yields in industrialized countries have reached a ceiling and conclude that this is not the case; they consider how yields may increase both in the short and long term. They also explore the yield gap; what is actually achieved against the best benchmark for a particular crop in a particular region. In efficiently run operations in industrialized countries, the gap may only be 20 per cent, and there may only be weak economic incentives to improve yields. However, there is considerable variation among different farming operations, with some—for complicated social and economic reasons—being very inefficient, and of course great scope for yield increases in many developing countries, especially through increased crop fertilization.

Jaggard and co-workers also review attempts to predict crop yields in the future by combining different types of global climate models (GCMs) with crop growth models. Results to date are informative but not consistent. The nature and extent of CO_2_ fertilization (see also Gornall *et al*. 2010) and in general how crops respond to climate change is insufficiently understood, and while GCMs tend to agree broadly about how increased greenhouse gas concentrations will lead to rises in temperatures, there is less agreement about which regions will get more or less rain, something that is particularly critical in predicting yields. The authors select a series of crops in 17 different countries and ask whether the goal of producing substantially more food in 2050 is feasible, given reasonable assumptions about rates of technological advance, efforts to close the yield gap, climate, CO_2_ fertilization and (often ignored) ozone pollution. Their conclusion, hedged with important caveats about the challenges ahead, is cautiously positive.

How might we increase the supply of meat and milk to match burgeoning demand? A variety of strategies are explored in the review by Thornton (2010). Conventional animal breeding is still capable of increasing yields, and will be important in addressing other goals such as sustainability and better welfare. Modern genomic approaches to breeding will undoubtedly produce further gains, perhaps supplemented by the prospect of genetic modification. Many advances have involved novel crosses, and preserving rare breeds may be a valuable investment for the future. We have a much better understanding of animal nutrition than in the past, but further research is required to develop robust animal growth models to help optimize livestock production. Poor nutrition is a particular problem in developing countries, where livestock often represent a critical component of household and community capital. Thornton describes a series of important new ideas that are specifically designed to benefit the nutritional status of livestock kept by very poor communities. In developed countries there has been a general decline in endemic diseases, although major epidemics, including new emergent diseases, continue to be a major threat. Less progress has been made in the tropics, although with some success such as the probable eradication of rinderpest. Animal breeding and veterinary advances, as well as better diagnosis and surveillance, will all help farmers keep pace with evolving pathogens and hopefully reduce the burden of disease.

Livestock production is responsible for a significant but contested fraction of anthropogenic greenhouse emissions (e.g. FAO 2006) and will be required to contribute towards mitigation efforts. Switching from ruminants to monogastric livestock may help, as will technological advances in how intensively maintained animals are reared. In more extensive systems it may be possible to develop systems that both capture more carbon and provide more feedstock. Maintaining viable livestock production will be critical in climate-change adaptation, especially for very many poor smallholders whose animals are central to their livelihoods. In some of the most marginal agricultural areas we may even see an increase in pastoralism and nomadism if crop production becomes unviable.

The supply of fishery products caught from the world's coasts and oceans has historically been a key source of high-quality food, and including the fish used for animal (and fish) feed, plus seaweeds; it is still the most significant component of the global aquatic food industry. It is also a highly complex and potentially vulnerable food system, consisting of a mix of industrial operations with significant political influence and small-scale or artisanal fishing, which provides an important source of direct food security and an income safety net for poor people. The major growth in marine fishery capacity over the last 50 years has resulted in almost all of the world's stocks being harvested to full capacity or over-exploited, with troubling implications for ecosystem health, stock resilience and long-term output and value.

Garcia & Rosenberg (2010) explore the future of marine fisheries, arguing that there is little scope for increased production, though a real risk of further declines in catches exists if overfishing continues. Over the last 40 years, the capacity of the global fleet to catch fish has increased sixfold and as actual harvests have remained nearly static, harvest productivity has thus declined by six. Critical for the future health of the sector is better governance, both for high-seas fisheries and those in Exclusive Economic Zones. There are particularly complex governance issues for fisheries exploited largely by poor people working in small groups. A better harmonization of fisheries and ecosystem management will help protect stocks and there is an important role for non-governmental agencies and civil society to champion sustainability in marine fisheries in order to help governments make difficult decisions, which may have unpopular political and socio-economic ramifications.

Inland capture fisheries—the fish and crustaceans harvested from rivers, lakes, floodplains and lagoons—are of major importance to many communities, especially those in low-income nations. Around 15–20% of global aquatic food is produced in this sector, though there is a widespread recognition that its significance to the economy and food security is underestimated because of under-reporting and the dominance of small-scale fishing operations. In low-income countries the food obtained from inland waters tends to be of direct importance as food, while in more wealthy counties recreational fishing has come to dominate.

Welcomme *et al*. (2010) explore the threats and opportunities for inland capture fisheries. The most important challenges involve changes to water systems with increasing demands for water from the agricultural, domestic, industrial and energy production sectors. Climate change is likely to exacerbate the problems. Integrated water management policies are essential to try to balance these competing interests. A range of management, mitigation and enhancement strategies are available, for example, enhancement of wild stocks by reared fish, but their success depends critically on the political and institutional context of the particular fishery. There is a continuum between unmanaged capture fisheries through increasing interventions to full aquaculture, and while aquaculture is often seen as an important development option, the transition from being a fisherman to working in more farming-like aquaculture can be a major barrier.

The development and growth of aquaculture has been one of the most remarkable features of the modern food sector, with production rising steadily in most parts of the world, increasingly supplanting capture fisheries as the most important source of fish and other aquatic food. As Bostock *et al*. (2010) discuss, there is considerable potential for expansion, with the major limiting factors being access to land and water as well as adequate market prices to provide a viable return on investment in installation and operating costs. Fish-based meals and oils from industrial fisheries have been used extensively as feed in aquaculture which raises issues of security of supply and environmental impact. However, dependence on this type of feed is likely to decrease with a wider range of species being cultivated, especially those from lower trophic levels (non-carnivores). Not unexpectedly, a large part of recent investment and technical development has focused on higher value species, though the real costs of former luxury species such as salmon has declined considerably. Looking ahead, we can expect to see a marked increase in aquaculture and product development involving lower-cost species, especially in low- and mid-income countries. Research challenges include increasing resource-use efficiency (requiring both biological and engineering innovation), reducing the risk of disease, improving environmental performance and maintaining the nutritional quality of farmed fish.

An often overlooked element of the food supply is ‘wild foods’—the plant and animal material that people harvest from non-agricultural ecosystems. Wild food is collected in developed countries but it is chiefly in low- and middle-income countries that it can be critical for a healthy diet. Bharucha & Pretty (2010) comprehensively review a large and scattered literature on wild food and find that a remarkably large number of species are used by different communities. For example, a meta-analysis of over 20 African studies finds that a typical rural community uses approximately 100 wild species of plant. In addition, most rural communities intervene to manage populations of wild species, suggesting that there is not such a sharp dichotomy between agricultural and non-agricultural systems.

Wild food is an ecosystem service provided by non-agricultural environments. As is discussed further in the review by Power (see below), it is important that these non-monetarized benefits of natural and semi-natural environments are understood in order to make rational decisions about land use at a time of increasing pressures on biodiversity.

## 4. Climate change and resource competition

Agriculture is globally one of the greatest consumers of water and, as Strzepek & Boehlert (2010) discuss, shortages of water may have a major effect on food production. Agriculture competes with domestic and commercial (municipal) consumers, and also with large-scale industrial users. The demand from both these sectors is currently increasing, roughly in line with growing global levels of prosperity (though water demand eventually plateaus in high-income countries). Water is also required to maintain functioning ecosystems and environmental flow requirements (ERFs) are not traditionally included in water calculations. Sufficient environmental flow is critical for freshwater ecosystems (and inland capture fisheries), but also for some terrestrial ecosystems: ‘blue’ water in lakes and rivers is hydrologically linked to ‘green’ (also called ‘brown’) water in soils. Future water supply will be strongly influenced by climate change, not least because evapotranspiration occurs at a faster rate in a warmer climate. Though climate change in some areas will be associated with higher precipitation, if this occurs as extreme events (storms and blizzards) it is of less use because of flooding and run-off. The world has also been using up groundwater reserves at a rate far above replenishment, a particular concern in shallow aquifers connected to surface hydrology.

Strzepek & Boehlert use a watershed level, global model to explore the water available for agriculture under different climate change scenarios. They conclude that meeting EFRs is perhaps the single greatest challenge to agricultural water supply, though there are huge regional variations—water dynamics are much more local than CO_2_ dynamics. Maintaining ERFs in watersheds such as the Colorado, Nile and Murray-Darling will be particularly difficult without affecting agriculture. At least until the second half of this century, water supply for agriculture will be much more severely affected by competition from other sectors than by climate change. Clearly, increasing water-use efficiency both in food production and in other areas is an important priority.

For many centuries humans could respond to increased demands for food by bringing more land into agriculture but as Smith *et al*. (2010) show, this is less of an option today. As populations grow and urbanization increases, more land is required for cities, and there is growing demand for non-food products such as fibre and wood. Any further encroachment of agriculture into natural habitats is a major threat to biodiversity, and limiting deforestation or other land-use change that leads to greater emissions is critical to reduce the rise in concentration in greenhouse gases. The threat of climate change has led to a major increase in the fraction of land used for biofuels, despite serious doubts about whether first-generation biofuel production actually results in a net reduction of greenhouse gas emissions when the consequences of this activity are fully accounted for.

Relatively little new land has been brought into agriculture over the last 50 years (though even this relatively modest conversion has often had major biodiversity impacts or affected the livelihoods of poor and indigenous groups), and the majority of the gains in production over this period have been due to improved yields. There is little scope for agricultural land expansion in Asia and most of Europe, but there may be considerable room for expansion of agricultural land use in South America and some room in sub-Saharan Africa. However, there would be significant environmental and cultural costs to future land conversion, especially if it involves the further destruction of rainforest.

Natural ecosystems provide a variety of services to mankind that are seldom included in traditional economic calculations (the benefits of marine and freshwater ecosystems through capture fisheries being exceptions). Power (2010) shows how an explicit consideration of these ecosystem services can help better manage the whole environment. Natural ecosystems often assist in reducing erosion and improving the nutrient content of soils, as well as regulating hydrological flows—all of value to farmers. They may also maintain healthy populations of pollinators and the natural enemies of weeds and pests, and hence increase yields in adjacent farmland. Agricultural land is also an ecosystem, albeit one strongly influenced by man. It too can produce positive ecosystem services, if managed appropriately, for example, flood control, wildlife habitats and carbon sequestration. Of course, agricultural production can also provide negative ecosystem services or, as Power refers to them, ecosystem disservices, for example, through pollution and biodiversity loss.

The ecosystem services approach is important in providing a broader perspective on the value of natural capital and understanding the full societal costs and benefits of different forms of food production. It is particularly helpful in efforts to try to incorporate within the food system the positive and negative externalities of food production, as well as identifying ‘win–win’ strategies, for example, agricultural practices that boost yield and increase sustainability. However, the economic theory underlying ecosystem services is still in its infancy and needs further development to increase its usefulness as a quantitative tool in decision-making.

The world is inevitably committed to some increase in average global temperatures between now and 2050. Gornall *et al*. (2010) explain that most climate models predict rises in temperature that will have mixed effects on agricultural production, possibly positive in medium latitudes but negative in the tropics, especially in areas where agriculture is already at the margin. Changes in rainfall patterns are harder to predict, and different regions will experience both higher and lower precipitation. But as Jaggard *et al*. (2010) also note, even where higher rainfall may benefit agriculture, if it occurs in high-intensity events much may be lost to run-off. Extreme events in general are likely to be an increasing problem for food production, with droughts, high temperature extremes, floods and (more controversially) tropical storms all likely to increase in frequency. Climate change will also affect food production through rises in sea level that risk inundating coastal agriculture, reductions in glacier cover that might drastically change the hydrology of rivers critical for irrigating large agricultural areas, and possibly through increases in pest and disease incidence, though the latter is very hard to predict accurately.

However, the effects of climate change are not all negative: increased CO_2_ levels can increase yields, especially in C_3_ plants. Indeed, when the Intergovernmental Panel on Climate Change (IPCC) estimated the effects of climate change on yields, it predicted modest global increases (IPCC 2007). There are, however, important caveats. First, the increase critically depends on CO_2_ fertilization, and several recent studies have suggested that this effect may have been overestimated (Ainsworth *et al*. 2008). Indeed, exactly how crops respond to the different components of climate change is a major area of uncertainty. Second, the global average hides winners and losers—many areas, particularly in the tropics, are likely to suffer radical yield reductions. Finally, even when regions benefit from, for example, longer growing seasons, these benefits can only be realized if the necessary well-adapted crop varieties, livestock breeds and human knowledge and expertise are available.

Agriculture affects emissions of greenhouse gases both because it requires inputs and energy derived from burning fossil fuels, and because it is a major emitter of greenhouse gases. Overall, agriculture uses a relative modest 3 per cent of global energy consumption, a major fraction of which is used in the production of nitrogen fertilizer. Energy use by agriculture will in the future be affected both by changes in energy prices as well as by the introduction of incentive mechanisms to reduce on-farm energy use. Predicting energy prices is hugely difficult but scenarios under consideration range from $50 a barrel to $130 or higher; as Woods *et al*. (2010) explore, prices at the high end would have major effects on the type of crops grown, their yields and the pressure to convert new land to agriculture. Incentives to improve energy efficiency are likely to include much more on-farm biomass production and energy generation.

It has been estimated that agriculture is responsible for about 20 per cent of all greenhouse gas emissions and is responsible for a major fraction of the anthropogenic production of methane and nitrous oxide (Stern 2007). Further emissions occur indirectly though land clearance for food production. Woods and co-workers explore opportunities to reduce these emissions, for example, by changed tillage practices, more efficient fertilizer use and production, and by agro-forestry schemes.

## 5. Cross-cutting themes

Over the last 50 years, the world has seen sustained growth in trade, including that in food products. The globalization of the food system has occurred owing to cheaper transport and communications, but also because of reductions in trade barriers and agricultural tariffs. Anderson (2010) explores these trends, and shows how developed countries continue to subsidize their agricultural sectors, though at rates that have declined significantly from a peak in the mid-1980s. Developing countries have historically exploited rather than subsidized their agricultural sector, though taxes and other burdens on agriculture are declining and recent estimates suggest a net subsidy in the developing world, albeit at rates still far below those in rich countries. A further major component of globalization is liberalization in the rules governing foreign direct investment, a trend that is promoting the consolidation of the private sector (retail, processing and agri-business) into many fewer global players.

The spate of trade restrictions prompted by the 2008 food-price spike and the failure to reach a multilateral trade deal that year shows that increasing trade liberalization is not a foregone conclusion, and indeed one possible future scenario is for developing countries, as they become richer, to impose their own subsidies and trade restrictions. Anderson reviews economic models that suggest that this will harm the poorest countries and increase volatility. Other factors which may influence global food markets are the evolution of the structure of the private sector, and the uncertainties associated with competition for energy (especially through oil prices and biofuels demand) and water, and the effects of climate change.

Food price volatility has long been a feature of agricultural markets and Gilbert & Morgan (2010) argue that recent fluctuations are not out of keeping with historical patterns. Volatility in most commodities has actually been lower in the past two decades than in the 1970s and 1980s, though rice remains a significant exception, perhaps because such low volumes are traded on global markets. Price fluctuations most often result from shocks to production or consumption, with extreme weather events and major civil disturbances being the most common drivers—though there are interconnections between financial and food commodity markets that are still not well understood. The impact of volatility on countries varies depending upon whether they are net food exporters or importers. For an individual household, the greater the proportion of income spent on food, the greater the adverse impact of food-price spikes, as was illustrated by the food riots in a number of low-income countries in 2008. Price spikes can rapidly become a major issue in domestic politics.

Given the difficulty of predicting future average food prices, it is not surprising that forecasting volatility—the fluctuations around the average food price—is even harder. The authors find no evidence that volatility is higher now than in the past. Looking ahead, the increased frequencies of extreme events with climate change will likely increase volatility while a more globalized food system can on the one hand buffer local perturbations but on the other propagate shocks more systemically. Volatility will also be influenced by changes to the governance of the global food system, for example, the possible introduction of real or virtual grain reserves that a number of groups have called for in the wake of the 2008 price spike (noting that others have argued that such interventions could be at best ineffective and at worst counter-productive in disincentivizing any supply response).

Piesse & Thirtle (2010) explore the relationship between investment in agriculture (research and development, technology and extension) and growth in yields and other measures of productivity. Since the Second World War, food production has kept pace with demand, due largely to scientific and technological innovation, first in developed countries, but critically then with transmission to developing countries (the Green Revolution). There is evidence that the rate of increase in yield growth is declining, though Piesse & Thirtle point out that if productivity is measured in terms of returns on total inputs beyond capital and labour (total factor productivity), this slow-down is less apparent. However, with overall investment in agriculture declining, in part, a result of low prices, continued innovation-led growth is far from certain.

Recent decades have seen both a decline in total investment in research, as well as a switch from the public to private sectors. It has also seen the transition of countries such as Brazil and China to become major investors in agricultural research and investment. Private sector investment inevitably leads to more restrictions on intellectual property and the authors are concerned that this may limit the transmission of new technology to low-income countries, as well as to less focus on the needs of the poorest countries.

To plan ahead it is important to have a forecast of future global food needs under a variety of different assumptions or scenarios that are as accurate as possible. This is difficult as the food system is complex, with dynamics determined by a combination of physical, biological and socio-economic processes. Reilly & Willenbockel (2010) review a number of exercises, which have sought to explore alternative futures. Most have adopted one of a variety of types of scenario analysis, combining different assumptions about exogenous driving factors such as population, global economic growth and climate change with an endogenous economic modelling engine. The economic models are typically partial equilibrium models, where the prices of different types of food in different countries connected by trade are determined by specifying such things as how demand is driven by personal income, supply driven by likely changes in climate, etc. Assumptions are also made about endogenous productivity growth.

The studies reviewed are generally relatively optimistic about the task of feeding a global population of 9 billion, though most predict increased food prices and require trade stability to match supply and demand in different geographical regions. Weaknesses include rather rudimentary model validation and deficiencies in the accuracy of the data used to initialize the models. More work needs to be put into coupling economic, biophysical and climate models. Economic models by their nature consider commodities traded on global markets and new approaches are required to explore more accurately the consequences of different futures for hunger and environmental sustainability. Nevertheless, despite these shortcomings, such models remain our only tool to try to explore the synoptic behaviour of the food system.

An important way to increase food supply and to decrease the environmental consequences of current food production is to reduce waste. It is often stated that between 30 and 40 per cent of food is wasted throughout the food system, though as Parfitt *et al*. (2010) point out, the evidence base upon which these estimates are calculated is weak, particularly with regard to losses in the developing world. There is also little data on the price elasticity of food waste: that is, how much of the problem would go away if food became more expensive?

Food is wasted at all stages of the food chain, from production and harvest all the way through to post-purchase by the consumer. In developing countries the highest losses occur at the post-harvest stage, typically owing to such factors as spillage and spoilage brought about by inadequate transport and storage infrastructure. Lack of capital for investment and poorly functioning socio-economic institutions hamper waste reduction, though Parfitt *et al*. suggest that waste in traditional or small-scale agriculture may have been over-estimated. In industrialized countries, substantial waste occurs in households after purchase, though retail, distribution and processing are also responsible for significant amount of waste; there is a marked lack of data on the magnitude of waste in catering and public food outlets. A reduction in waste early in the food chain has occurred in many countries as they move from low- to middle-income status, though these gains are sometimes mitigated by increasing waste by consumers and the retail trade. Encouragingly, a variety of targeted incentive schemes in high-income countries have demonstrated significant potential for waste reduction, given the political will.

Food security and health are closely intertwined, as discussed by Hawkesworth *et al*. (2010). They begin by describing the basic nutritional components of a healthy diet and how deviations from this can lead to malnutrition and hunger, or obesity and its associated health problems. As also discussed by Kearney, the health services of many middle-income countries are having to cope with the double burden of under- and over-feeding. Hawkesworth *et al*. highlight the many gaps in our knowledge about people's diets, especially in developing countries, which hamper better policy development. They argue that reliance on indirect proxies such as those based on the balance of a country's known or estimated production, imports and exports, can be seriously misleading and they champion the importance of household and individual surveys based on measuring consumption rather than availability. There have been significant improvements in collecting data on poverty to address the millennium development goals, which may provide a model for getting better data on diets.

## 6. Conclusions

The reviews published here describe the size of the challenge facing humanity in maintaining *per capita* food production and in trying to reduce the number of people who suffer hunger and malnutrition. They provide a number of grounds for optimism: the high likelihood of population and consumption demand reaching a plateau some time during this century, and the major opportunities for yield growth through the application of traditional and novel science. They also stress the significant yield gains that can be obtained by working closely with farmers and fishermen to deploy existing knowledge and resources through better education and greater social and economic equality—closing the gap between realized and current possible levels of productivity and improving efforts to reduce waste. Another theme that emerges is the importance of taking a ‘competing risks’ approach to regulation in the food system—it is too easy to close off options by applying naive versions of the precautionary principle. The world is going to have to produce more food, and unless much of the Earth's remaining biodiversity is to be destroyed, this will need to be done without expanding the area under cultivation. Achieving higher yields from the same acreage without severely impacting the environment requires a new way of approaching food production—sustainable intensification (Royal Society 2009; Godfray *et al*. 2010). The reviews also highlight the importance of treating food production in a broader context, as one of several major competitors for freshwater, land and energy, and as integral to the world's overarching challenge of mitigating and adapting to climate change. Finally, they emphasize the numerous research questions that require investigation, not just in the natural sciences but in economics, politics and the social sciences.

In the eighteenth century, Thomas Malthus alerted the world to the consequences of rapid (geometric) population growth for what we (though not he) would call food security. Thanks to the demographic transition, we can now envisage a world where population and consumption cease to rise. The policy decisions we make and the science we choose to carry out over the next few decades will determine whether all the people living in this world have access to adequate food.

## Endnote

1 The views and opinions of the authors in this volume and this overview article do not represent the views of the Government Office for Science or the UK Government.
